# Remarks on Negro Consumption

**Published:** 1840-12

**Authors:** A. H. Buchanan

**Affiliations:** Columbia, Tennessee


### WESTERN JOURNAL

OF

### MEDICINE AND SURGERY.

DECEMBER, 1840.

Art. I.—*Remarks on Negro Consumption.* By A. H. Buchanan, M. D., of Columbia, Tennessee.

There prevails a disease in Tennessee known as Negro Consumption, which annually proves destructive to many of our black population. The character of this disease is by no means generally understood, and we are frequently asked, what is the nature of this malady? Is it peculiar to the negro? Why is it called "*Negro* Consumption," &c.? Not long since in consequence of this name, "negro consumption," not being known in the books as designating any known affection, we were called on for our deposition in a medico-legal point of view, touching the nature of this disease, in a case involving property to some amount. The following are some of the questions put by the attorney: What is negro consumption? What are the symptoms by which you judge of the existence of this disease? Is it peculiar to the negro? Is it the same kind of disease known by the name of phthisis pulmonalis, or consumption among the whites? What causes produce the disease? Is it a curable affection? &c. &c. Now all of these questions, according to the answers given, it can readily be seen, an ingenious lawyer might wield with great effect in his pleadings. But it so happened in the above case that the parties came to a compromise, and we were spared from any further questioning. I shall endeavor however to answer some of these questions, with a view of defining our meaning of the term "negro consumption;" and to describe its nature, shall also give the pathological appearances of some of the cases that have come under my observation. By negro consumption we mean a scrofulous affection which is principally fixed upon the deep seated lymphatic glands, the viscera of the thorax and abdomen, and upon the serous membranes lining these cavities; it is rarely the case that the superficial lymphatic glands of the neck, axilla or groin, are affected in this disease, and we are hence not often aided in our diagnosis by an examination of these parts. The disease consists essentially in an extensive tubercular disorganization of the viscera of the thorax and abdomen. The symptoms of this disease are not very prominent, but to one who has been in the habit of observing the general appearance and movement of those affected with it, they are sufficiently so to characterize it; and towards its close with much certainty. In the commencement, however, the symptoms are particularly obscure and doubtful, so much so, that the individual himself scarcely recognizes any alteration in his feelings. He has sufficient appetite, and performs his usual daily labor, and except for a sense of languor and debility, and some slight uneasiness in the chest and abdomen, he feels so well that he would make no complaint. But these symptoms gradually increase, perhaps for several weeks, when his spirits become affected, and with a dull inanimate countenance he still lingers on at work, performing his task with great difficulty: he now begins to attract his master's attention, and he is sent to his cabin, where he remains for several days without exciting any uneasiness about his recovery, for he eats heartily and does not complain except of some slight pain in his chest and abdomen, of weakness and of shortness of breath; but he is entirely unable to work, and has taken up the idea that he is poisoned, (and hence the name of "negro poison,") for it is an idea that invariably infests the minds of all those whom I have examined, whose age renders them capable of reflection. The master also is not unfrequently impressed with the same opinion, and attributes his disease to some article of a poisonous nature administered by a neighboring enemy. It is about this period of the complaint that the physician is sent for; or, as is often the case, that the patient is sent to the physician, and it is now that we can recognize the complaint at once; the symptoms have become sufficiently marked and decided to characterize it with great certainty. The skin is dry and ashy, and the countenance desponding. There is great muscular debility and inactivity, with slight emaciation, and always more or less pain in the chest and abdomen, accompanied with difficult respiration, or as they forcibly express it, "shortness of breath;" this last symptom is particularly manifest if they are hurried out of a walk, or caused to ascend a flight of stairs or other ascent. The tongue is generally pale and covered with a whitish fur, though seldom thickly coated; the pulse, if examined while the patient is in an erect position, is quick, hurried, and somewhat tremulous; in the sitting position much more natural; if the patient is made to walk to and fro quickly, the pulse is very much agitated, quick and tremulous; and the heart is often felt to throb and beat with force against the ribs, while the patient literally pants for breath. In some instances the sclerotica may now be observed to be of a pearly whiteness; the appetite remains good, and sometimes even voracious to the very last; the bowels are readily acted upon by medicines, and sometimes they are troubled with diarrhœa, particularly towards the close of the disease; in some instances there is a slight hacking cough with slight expectoration of mucus, and preternatural heat of the skin, with a slight febrile action of the pulse. In the last stages of the complaint, a dropsical effusion takes place into the cavities of the chest and abdomen, and not unfrequently in the pericardium, and sometimes extends to the lower extremities. In some instances this dropsical affection occurs earlier in the complaint. The patient at this time and even before, is particularly prone to sit for hours together, doubled up and leaning forwards with the elbows resting upon the knees, or the elbow upon the knee supporting the head, and to the last hour of life most of these patients prefer the sitting position. The next question, "Is this disease peculiar to the negro?" I unhesitatingly answered no; because I regard it as a scrofulous or tuberculous affection to which all the human race, as well as various other animals, as the horse, ox, sheep, birds, and even insects, are liable: but certainly the black race is much more predisposed to that form of the disease which produces the above symptoms than our white population; so much so, that among us it has taken the peculiar name of "negro consumption;" and I am certain that until very lately, I have never met with a case among our white population that fully characterized this complaint. That scrofulous affection which is principally confined to the mesenteric glands and called *tabes mesenterica*, I have occasionally met with in our white race, as well as that called *king's evil* which principally shews itself in the superficial lymphatic glands, and also other scrofulous affections, as of the joints, eye, &c. But that deep seated and extensive scrofula of all the vital organs of the great splanchnic cavities which is so common and fatal among our black population—sparing neither age nor sex, and in some instances carrying off whole families in a few months, is rarely met with among the white race. This disease, therefore, though not peculiar to the black ruce, is perhaps with sufficient propriety called "negro consumption."

"Is it the same kind of disease known by the name of phthisis pulmonalis or consumption among the whites?" This question cannot be so positively answered; it is one upon which the most learned pathologists have differed and still differ. The question in another shape is, "are all tubercular diseases of the same nature" In the disease above mentioned, (as will be shown presently,) we have that diseased condition of the lymphatic glands which is called scrofula, and that condition of the lungs, liver, spleen, &c. which is called tuberculous disease, and therefore two diseases; or else what Dr. Duncan, Parr and others affirm on this subject must be true—"That each tubercle is a lymphatic gland in a diseased state, the consequence of scrofula;" and we have but one disease, namely, scrofula. Or else, if we have two diseases, they co-exist, increase and disorganize in the same manner, and at the same time, different organs and tissues of the same individual, by a peculiar *deposition* the physical properties of which, when examined from a lymphatic gland, cannot be distinguished from those taken from another organ either by the anatomist or chemist. Such being the fact, it is fair to conclude that the same kind of diseased action, which produced the deposition in the lymphatic gland, also produced it in the other organs and tissues; and therefore, that in the disease under consideration, where the same appearances of destructive disorganization occur simultaneously in the lymphatic glands, lungs, liver, spleen, and in the serous, mucous and cellular tissues, all such appearances are produced in each of the organs and tissues by the same disease, whether it be called tuberculous or scrofulous. If then these affections are identical in the black man, they must be so in the white man, and if the same in one that they are in the other, they must be the same in each, wherefore I must believe that the "negro consumption" is the same kind of disease, called tubercular phthisis or consumption in the white man.

I am aware that I have but expressed the opinion of very many of the most distinguished pathologists, in the above conclusion as to the identity of scrofulous and tubercular diseases. The following extract from a "Treatise on tubercular phthisis," by Dr. James Clark, expresses the opinion of the majority of modern pathologists on this subject: "From remote antiquity to the present day, the disease of which the present matter (tubercular) constitutes the destructive anatomical character, has received different names according to the development in particular organs and tissues. In the external glands and in the bones it is commonly called scrofula; in the lungs, phthisis; and in the glands of the mesentery tabes mesenterica, &c. The identity of these affections was only suspected by the ancients from the similarity of the general symptoms, but has been demonstrated by the moderns on the clear evidence of morbid anatomy; an increased attention to which science and the study of the causes of the disease has led pathologists to entertain more accurate opinions, and more comprehensive views regarding it." But notwithstanding these sentiments it is well known, that this is by no means a settled point in pathology, and that many of the most intelligent entertain different views, and regard it as extremely difficult, if not impossible to "identify the tuberculous with the scrofulous diathesis;" (Morton, Phila.,) but let this be so, and still the identity of "negro consumption" and tubercular phthisis, cannot be denied, for we have the same kind of tubercular disorganization of the lungs in both cases.

The next question, "What causes produce the disease?" necessarily gives rise to another; Is the disease hereditary? I believe that it is; first, because I know of some instances, and have heard of others, where all the members of the same family have perished of this affection, while the remainder of the slaves on the same plantation have escaped, notwithstanding they were exposed to the influence of the same circumstances, fed, clad, worked, and otherwise treated in the same manner, and exposed to the same weather. Second, because I believe it is of the same nature of tubercular phthisis, which all the world admits to be hereditary. An hereditary disposition, or constitutional cachexy therefore, we regard as the principal predisposing cause of the disease; and as to the question, also asked, "Does the disease ever originate in an individual who is not predisposed at birth?" I am of opinion that it does, or at least that it may; for according to Dr. Clark the predisposition "may be acquired at any period of life, from infancy to advanced old age" by the long continued operation of all those circumstances, "which debilitate and increase the irritability of the system, impede the due digestion and assimilation of the food, diminish the various secretions and excretions, and increase internal sanguineous congestion." But whether this predisposition exists as an inheritance from birth, or is acquired in the course of life, it is well known that it is not deemed by all as essential to the development of tubercles. Broussais and his followers, and many of the most distinguished pathologists of the present day, among whom stands conspicuous Professor Gross, of Louisville, believe that chronic irritation or inflammation may produce tubercles, in any of the tissues or in any individual, however sound may be his constitution at the commencement of diseased action; but of course where the predisposition exists the disease is much more readily produced by the operation of any of the exciting causes, and I am therefore fully sustained in the opinion, (by both sets of pathologists,) that the disease may originate in any individual.

The exciting causes of this disease are numerous, and are in general all those which are enumerated in the books as exciting causes of phthisis; but of all these the most operative in the production of this complaint is exposure to cold, to damp and variable weather. This I infer from the fact, that our black population are well fed on wholesome food, and get enough, *at least*, of the light and heat of the sun, and of pure air to prevent the formation of tubercles; but they are not in general sufficiently clothed to resist the depressing influence of extreme cold, and the sudden changes of weather to which they are almost constantly exposed. In a climate like ours, in Tennessee, where the vicissitudes of temperature are so sudden and so great as not unfrequently to produce a range in the thermometer of 40 and 50 degrees in a day; and where the winds with equal uncertainty shift from the gentle and relaxing breeze of the south, to the freezing and benumbing blasts from the north; and where the bright blue skies and cheering sun are suddenly obscured by clouds, and heavy and repeated rains for days together to the amount of six, eight, and even ten inches per month, as has been the case this season, producing a heavy humid atmosphere for weeks at a time; where all these changes are thus constantly going on, it is evident that the clothing should be adapted to the changes of the weather. But what is the fact? The negro has but one suit of clothing at a time, and with this while it lasts he meets all the various changes that may occur in the humidity or temperature of the atmosphere, and whether it be in the summer season or the winter season, he never dreams of flannel next his skin, or over-shoes, nor muffles up in his cloak, to protect himself from the frosts and chilling blasts of winter; but with his cotton shirt and linsey roundabout and drawers he buffets all the storms of the season. Who, therefore, among us can doubt the exciting cause of negro consumption? A race whose congenial clime is upon the burning coasts of Africa, and who delight to bask in the rays of a meridian sun, to be thus exposed in a climate unsuited to their nature, and with a high susceptibility to the influence of *cold*, must of necessity fall victims to its influence.

We come now to the next and last question of our attorney, "Is it a curable affection?" We answer, No!—it is a perfect approbium medicinæ. In the form of tubercular consumption, it carries off one-fourth part of the inhabitants of all Europe, (Dr. Young,) and the annual victims to its ravages in Great Britain alone, was estimated by Dr. Woolcombe at fifty-five thousand, and even in our own happy country, one-fifth of the mortality of our large cities is produced by it. (Emerson.) Go therefore, clothe your negroes better and take more care of them, and *you* may *prevent* what *we* cannot *cure.* Thus we would speak to the owners of slaves.

In the course of the last five or six years we have had the opportunity of making autopsical examinations of sixteen cases of this disease, the pathological appearances in all of which were remarkably similar. In the first case, of a negro woman aged about 25 years, the head was not examined. Thorax—Immediately below the sternum I observed several small glands filled with crude tuberculous matter. The left lung was firmly united to the side from apex to base by granulated adhesions of the opposite surfaces of the pleura. On ncising the lung in different places we found it perfectly disorganized by tubercles in various stages of development. Small abscesses, granules and crude tubercular matter occupied all its substance; right lung firmly adherent in places, and the pleura and lung filled with tubercles; the left pericardium distended with serum; the heart rather soft and flabby. Abdomen.—On opening the abdomen I found the opposite surfaces of the peritoneum cemented together, highly inflamed and filled with tubercles; the intestines united by adhesions of false membrane filled with tubercles; the mesenteric glands greatly enlarged and filled with crude tuberculous matter. Liver—some small tubercles upon its peritoneal surface and in its substance. Spleen—several tubercles on its surface and two large abcesses in its substance. Kidneys contained tubercles. This woman sat up, and even walked about until the evening before she died; she ate cabbage and such other articles of diet for dinner, and went to bed as well as usual in the evening, but next morning was found dead in her bed. On opening the stomach it appeared sound and contained several articles of diet in a half digested state. I will here remark that in four instances of this disease, to my knowledge, death occurred in the same sudden and unexpected manner. One of these cases I examined, and found, in addition to the usual extensive tubercular deposits in the viscera of the thorax and abdomen, a highly diseased condition of the heart. The pericardium was filled with a reddish serum. The external surface of the heart had the appearance of being blistered, and around the apex was coagulable lymph of recent deposit; the muscular substance was very soft, so that the finger could be readily pushed through it. In this case disease of the heart was predicted, from the unusual throbbing and uneasy feeling about its seat, upon very slight exertion, and also from the very tremulous pulse, and sometimes almost pulseless condition of the wrist. I was assisted in this examination by Drs. Law and Davidson, of Columbia.

I will mention a circumstance connected with another case, which as it gave rise to much doubt as to the true nature of the patient's complaint, may possibly aid others in their diagnosis. A negro man, aged about 20 years, who had the usual symptoms of negro consumption applied to me for assistance, stating that he had tried others without benefit, and that he was poisoned—he was somewhat emaciated, with a quick and tremulous pulse, great debility, shortness of breath, and abdomen distended with a dropsical effusion; appetite tolerably good; but that which distressed him most was "something beating in his belly." Upon examination I found a pulsating tumour about the size of an orange, a little above and to the left of the umbilicus, but which could be very indistinctly felt at the time in consequence of the fluid in the abdomen. This tumour was thought by many to be aneurism of the aorta, and gave rise to much doubt in the minds of all. I regarded his case however as hopeless, and put him upon a mild palliative course of treatment; but with this he soon become dissatisfied and in a few days applied to a Root Doctor, who agreed with him that he was poisoned, and treated him for *poison* several weeks, during all which time he become more and more emaciated. He at length applied to a Steam Doctor by whom he was very speedily despatched. I accidentally heard of his death, and Dr. A. H. Brown of this place and myself opened him in his grave two nights afterwards, and by moonlight dissected out the tumour, which we found upon examination to be a mass of tubercular matter in the mesentery, which pressed upon the aorta, with sufficient force to receive and transmit its pulsatory movements through the parietes of the abdomen. It is scarcely necessary to detail the appearances of another case. Certainly in all instances the tuberculous deposits are not so great as in the first case above mentioned, but it is a very fair representative of all. I will further mention that in all cases where the stomach was examined its internal structure appeared sound, but in all the instances, (six in number,) where I have examined the intestinal mucous membrane, it has presented extensive and deep ulcerations. In one case the ulcerations had perforated the serous coat in several places, but these perforations were prevented from opening into the cavity of the peritoneum by adhesions of false membrane and tubercular lumps, which projected into the cavity of the bowels. I was assisted in the examination of this case by Dr. Brown, of this place. In another case the duodenum, through its whole length, was dotted with small superficial ulcerations, the size of a ten cent piece or smaller; the coats of this bowel however were very much thickened and indurated, and in the remainder of the intestines were ulcers, but particularly numerous in the last twelve or eighteen inches of the small intestines. Dr. Law was present at the examination of this case. The last case of this disease which I examined was during the last summer. I rode twelve miles to see it in order to satisfy the mind of the master, who was under the impression that his slave was poisoned. The case was a negro man, aged about 35 years; I was accompanied by three or four students of medicine who were anxious to see the cavity of the abdomen and its contents, which I had promised to shew them. I made the common crucial incision through the parietes of the abdomen, but could find no cavity. The opposite surfaces of the peritoneum adhered so firmly, and the spaces between the convolutions of the intestines were so filled with lymph, false membrane, tuberculous, and dark colored matter, that the whole presented a mass of disease impossible to describe, and which time did not permit us to investigate. It would have taken hours of tedious and careful dissection to have even separated the abdominal parietes from the intestines. I was more lucky in showing the viscera of the thorax, for here I found them less diseased than is common. The lungs contained some tubercles and small abcesses. Heart sound. There were no remarkable symptoms in this case that authorized the belief of such extensive disease in the abdomen, and what is a remarkable fact, he bore pressure upon the abdomen without complaint. He suffered from ascites in the course of his disease, but the water was purged off some weeks before he died.

*Treatment.*—I have nothing to offer as a remedy for this complaint, every article that I have tried having seemed but to aggravate the symptoms, at least doing no good. Bleeding and purging are highly injurious as they prostrate at once, and counter-irritants, as blisters, setons, issues, and tartar emetic pustulations, have all been tried without any benefit. Tonics have had a better effect than all other remedies; but I have never known them to cure a case. Perhaps in the earliest stages of the disease, when unfortunately we are never consulted, some of the preparations of iron might be used with benefit.

*October*, 1840.

